# The Association between Residence Floor Level and Cardiovascular Disease: The Health and Environment in Oslo Study

**DOI:** 10.1155/2016/2951658

**Published:** 2016-12-07

**Authors:** Mads K. Rohde, Geir Aamodt

**Affiliations:** Department of Landscape Architecture and Spatial Planning, Norwegian University of Life Sciences, Ås, Norway

## Abstract

*Background.* Increasingly more people live in tall buildings and on higher floor levels. Factors relating to floor level may protect against or cause cardiovascular disease (CVD). Only one previous study has investigated the association between floor level and CVD.* Methods.* We studied associations between floor of bedroom and self-reported history of stroke, venous thromboembolism (VTE), and intermittent claudication (IC) among 12.525 inhabitants in Oslo, Norway. We fitted multivariate logistic regression models and adjusted for sociodemographic variables, socioeconomic status (SES), and health behaviors. Additionally, we investigated block apartment residents (*N* = 5.374) separately.* Results. *Trend analyses showed that disease prevalence increased by floor level, for all three outcomes. When we investigated block apartment residents alone, the trends disappeared, but one association remained: higher odds of VTE history on 6th floor or higher, compared to basement and 1st floor (OR: 1.504; 95% CI: 1.007–2.247).* Conclusion. *Floor level is positively associated with CVD, in Oslo. The best-supported explanation may be residual confounding by building height and SES. Another explanation, about the impact of atmospheric electricity, is also presented. The results underline a need to better understand the associations between residence floor level and CVD and multistory housing and CVD.

## 1. Introduction

Cardiovascular disease (CVD) constitutes a major public health burden and is the greatest cause of mortality globally. Stroke alone is the second greatest cause of death [1], while venous thromboembolism (VTE) is considered the third most common acute CVD [2]. Thrombosis is the most common underlying pathology of stroke as well as VTE [3]. Their pathogenesis differ, but studies suggest a common etiology [4, 5], since a large proportion of VTEs lack major provoking factors [6]. In addition to age [4], VTE may share risk factors such as smoking [7], obesity [8], psychosocial environment [9], air pollution [4], and socioeconomic status (SES) [10] with stroke [11–13] and other arterial CVDs, such as intermittent claudication (IC) [14].

With today's trends of urbanization [15], living in multistory buildings and on higher floors is becoming increasingly common and gives reason to be vigilant about potential floor level effects on health. Previous studies on the association between multistory housing and health and floor level effects have barely investigated health outcomes at all, except for mental health [16–18]. To the best of our knowledge, the association between floor level and CVD has been investigated only once. In a Swiss study, including the entire population of block apartment residents, Panczak and coworkers [18] found that stroke and general CVD mortality decreased by increasing floor levels. One study by Wolinsky and coworkers [19] found significant associations between living in multistory buildings and development of stroke in an elderly population but did not investigate whether incidence rates were related to floor level.

To date, four different explanations of an association between floor level and CVD have been proposed. Three explanations have been suggested by Panczak and coworkers [18] and would all likely produce a negative association: the vertical distribution of the physical factors of environmental noise and air pollution; physical activity levels by use of stairs; and an indirect effect related to the characteristics of individuals at different floor levels, for instance, in terms of socioeconomic status. A fourth and novel hypothesis [20] is derived from theory about the earth's natural electromagnetic environment [21, p. 25] and its variation with distance from ground level. According to this hypothesis, the association between floor level and CVD would be positive and thus diametrically different from the other three mechanisms presented above.

The aim in this study was to investigate the association between floor level and CVD morbidity. We used stroke as our main outcome variable. To better understand the influence of a late-look bias [22] caused by relocation of participants from higher floors after suffering from stroke, we include VTE and IC as health outcomes.

## 2. Materials and Methods

### 2.1. Study Population

**Figure 1:**
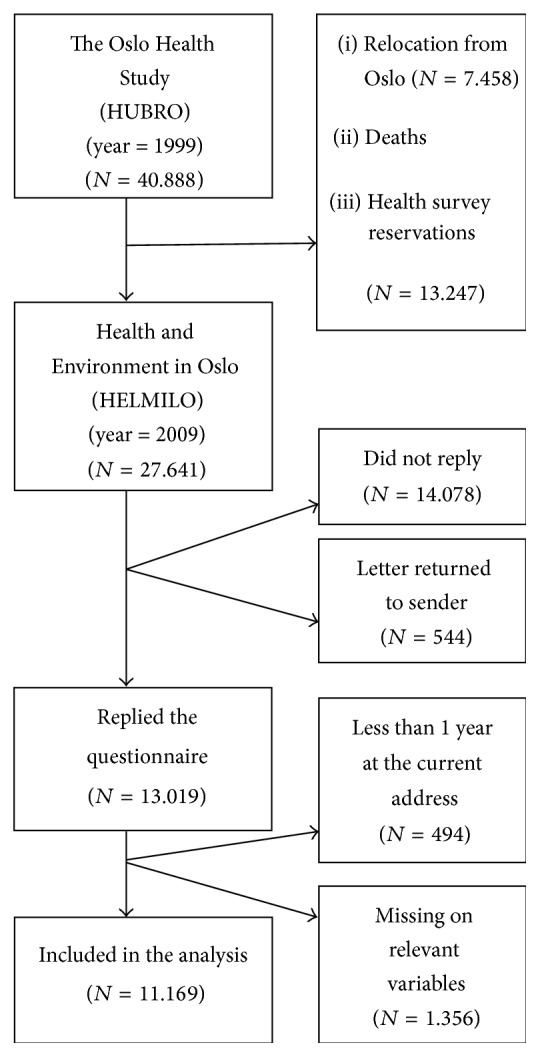
Overview of the sample.

We used the Health and Environment in Oslo study (HELMILO) which is a cross-sectional study of the inhabitants of Oslo, Norway, conducted in 2009/2010. The sample consists of five age groups between 39 and 85 years (born in 1924/25, 1940/41, 1955, 1960, and 1970). The sample had originally been drawn for inclusion in the Oslo Health Study (HUBRO) and encompassed all Oslo inhabitants in the chosen age groups, as registered in the Norwegian National Registry as of December 1999 (*n* = 40.888). HELMILO extends HUBRO with a particular focus on environmental factors. The size of the original sample was reduced to 27.641 in August 2009 (due to death, relocation from Oslo, and reservations against future health surveys), all of whom were sent the HELMILO questionnaire by mail. A total of 544 respondents were subtracted from the sample due to wrong addresses. With 13.019 completed questionnaires returned, the response rate was 48%. This is illustrated in Figure 1.

We excluded those who reported that they had lived less than one year at the current address (*N* = 494) and those with missing values on at least one of the study variables (*N* = 1.356). A total of 11.169 participants were included in our final analyses. All measures used in this study were self-reported, except for year of birth, country of birth, and gender, which were retrieved from the National Registry.

### 2.2. Variables

#### 2.2.1. Outcome Variables

The questionnaire contained the inquiry “Have you or have you had?” followed by a list of sixteen different disease outcomes. Response choices were “yes” and “no” as well as two additional alternatives of “yes” and “no” with an inscription above, “confirmed by a doctor.” The following three cardiovascular events were chosen for this study (our nomenclature in brackets): “stroke (cerebral infarction/haemorrhage, ministroke)” (stroke); “blood clot, phlebitis” (venous thromboembolism (VTE)), and “hardening of the arteries in the legs” (intermittent claudication (IC)).

Prevalent cases were defined as participants having or having had the disease, irrespective of whether the disease was reported to have been confirmed by a doctor. Those with missing values on both subquestions of disease (disease/doctor confirmed disease) remained missing in our new variable. An exception was the instances where values were missing at one disease inquiry, but complete on one of the other two disease inquiries. In these cases, missing values were interpreted as “no.” Participants who answered both “yes”* and* “no” on one of the subquestions of disease (disease/doctor confirmed disease) and at the same time left the other set unanswered or answered “yes”* and* “no” on both subquestions were coded as missing.

#### 2.2.2. Floor Level, Period of Residence, and Type of Accommodation

*Floor level* was retrieved from the question “On which floor is your bedroom? (0 = basement, 1 = 1st floor, etc.).” This information was reported for both present and previous residences. We utilized present floor level only. We kept the original variable, and we produced a variable with five categories, where we grouped the floors into the following categories: 0-1st, 2nd-3rd, 4th-5th, 6th–10th, and *⩾*11th floor.* Period of residence *at current address was given in three categories: “less than 1 year”; “1–10 years”; and ”more than 10 years,” from which we made a variable including only the two highest categories. Respondents reported to inhabit one of four* types of accommodation: *“detached house/villa”; “block apartment/terraced flat”; “undetached/semidetached house”; and “other residences.”

#### 2.2.3. Country of Birth and Living with Someone

*Country of birth* was given in 15 categories constituting different regions, which we categorized into three: Norway; Eastern Europe, Western Europe, and North America; and all other countries (Africa, Asia, South America, and Oceania). Whether the respondents were* living together with someone* was determined by the question “Do you live together with someone?” with answer alternatives “yes” and “no.”

#### 2.2.4. Socioeconomic Status (SES)

We included two available measures of socioeconomic status. Education level was reported as number of years of schooling. We split this variable into three education levels: 12 years or less; 13–16 years; and more than 16 years of education. Occupational status was initially given in nine categories at an ordinal scale. The participants chose one or more employment categories that they were or had been employed in. We used the highest status reported. In a new variable, we kept the first category: “administrative leader, politician.” Then, we collapsed the next two categories: “academic occupations (at least 4 years of high school or university education)” and “occupations with shorter high school or university education (1–3 years) and technicians.” A third category was produced from the following two occupational categories: “office and customer service occupations” and “sales, services, and care professions.” The fourth category, which we termed blue collar workers, constituted those who were or had been employed in “farming, forestry, or fishery occupations”; as “craftsman, builder, labourer, and so forth”; as “operator (machinist), driver, and so forth”; and in “elementary occupations without need for formal education.”

#### 2.2.5. Health Behavior Variables

*Body mass index* (BMI) was categorized into four categories using the borders set by the World Health Organization [23]: obese, overweight, normal weight, and underweight. Underweight respondents (*N* = 174; 1.6%) were merged with those of normal weight. Consumption of* fruit* (“fruits/berries”),* vegetables *(“vegetables/salad”), and* fatty fish *(“fatty fish”) was reported separately on a six-point ordinal scale. We collapsed the categories on this scale pairwise, into eating 1–3 times per month or less (low consumption); 1–6 times per week (moderate consumption); and at least once per day (high consumption).* Alcohol consumption *in the past twelve months was categorized into three levels from an original eight-category ordinal scale variable (our nomenclature in brackets): having never drunk, not drunk last year or only a few times last year (infrequent alcohol consumption); having drunk approximately once per month, 2-3 times per month, or approximately once per week (moderate consumption); and having drunk 2-3 or 4–7 times per week (frequent consumption).* Smoking status* was reported in three categories (present smoker, previous smoker, and never smoked).* Physical activity* was measured with the question “State your movement and physical activity in your leisure time.” It was emphasized that the question only concerned the last 12 months and that one should choose an average if the activity varied considerably, for instance, between summer and winter time. We kept the variable in its original five-level ordinal scale level version, with the categories being (ascending order from sedentary to very active) “read, watch TV or other sedentary activity,” “walk, cycle or move about in some other way at least 2–4 hours per week,” “walk, cycle or move about in some other way at least 4 hours,” “take part in sport, heavy gardening, and so forth (at least 4 hours per week),” and “exercise hard or take part in competitive sport regularly and several times per week.”

### 2.3. Statistical Analysis

Chi-square tests were used to investigate the association between categorical variables such as prevalence of the three disease outcomes and floor level. We used logistic regression to model the odds for the outcomes as a function of floor levels, with “basement and 1st floor” as reference category. In multivariate models, we controlled for potentially confounding variables. The covariates were included stepwise in three blocks of belonging variables. Results are reported for all three models, as well as crude estimates (Model 0). The variable* period of residence* was not included as a covariate, but an interaction term between the variable and floor level was included in the final model to test for the modifying effect of period of residence on the odds of having experienced one of the cardiovascular events. We tested for a trend of increasing prevalence of CVD by increasing floor level by implementing the floor variable at a continuous measurement level in each logistic regression model. The whole analysis procedure was repeated for block apartment residents only.

Analyses were conducted in SPSS version 22. *p* values less than 0.05 were considered as statistically significant.

### 2.4. Ethics

HELMILO is approved by the Regional Committee for Medical and Health Research Ethics, Norway. We used anonymous data in the present study.

## 3. Results

Population characteristics are reported in Table 1, showing how the participants are distributed dependent on which floor they sleep on. A greater proportion of elderly people (born in 1924-25) resided on the highest two floor levels (13.2% and 18.2%) than on the 0-1st floor (9.4%). The proportion living with someone was the highest on the lowest floor level (79.3%) and the lowest at the 6th–10th floor (54.2%). The proportion with nonwestern origin, the lowest level of education (≤12 years), and the lowest occupational status was higher on higher floor levels. More current smokers lived above the 4th floor (18.8–21.2%) than on lower floors (14.5–15.5%), the highest proportion of obese individuals lived on the 6th–10th floor (15.6%), and the most sedentary ones lived on the 6th floor or higher (about 15% versus about 10% on lower floor levels). From the 4th-5th floor upwards, the proportion of infrequent alcohol consumers steadily increased from about 16.5% on lower floors to 19.2% on the 4th-5th floor and up to 27.3% on the highest floor level.

The figures were similar among block apartment residents alone (data not shown). However, participants in block apartments living on the 0–3rd floor were, for instance, less educated and more often of nonwestern origin compared to the same floor levels in the full sample.

The floor level values had a range from 0 to 33, median and mode of 2, and mean of 2.33. Only 32 residents reported to reside above the 12th floor (data not shown). A total of 19.2% of residents who reported to live on the 11th floor or higher also reported that they did not reside in multistory buildings (data not shown). Parallel figures for those living on the 6th–10th floor were 2.6%.

In Table 2, we report the distribution of the prevalence of three disease outcomes and floor levels. The prevalence of stroke, VTE, and IC differed significantly across floor levels (*p* ≤ 0.001).

Results from the regression models are shown in Table 3. In the crude models, the odds of having experienced a stroke were significantly higher on the 6th–10th floor (OR: 1.578; 95% CI: 1.061–2.345) and the 11th floor or higher (OR: 2.5; 95% CI: 1.311–4.766) compared to basement and ground floor residents. The same was true for venous thromboembolism (VTE) (OR: 2.043; 95% CI: 1.415–2.951; OR: 2.332; 95% CI: 1.191–4.566). The odds of having experienced intermittent claudication (IC) were significantly higher among residents on the 11th floor or higher compared to individuals living in the reference category level (OR: 2.917; 95% CI: 1.597–5.327).

In the fully adjusted models, the effect measures were generally attenuated. The associations between floor level and stroke were no longer statistically significant. Residents on the 6th–10th floor had increased odds of having or having had VTE (OR: 1.720; 95% CI: 1.174–2.518) and residents on the 11th floor or higher had increased odds of having or having had IC (OR: 2.318; 95% CI: 1.237–4.345). The test of trend showed a statistically significant increase in prevalence of* all* CVD outcomes as a function of floor level, also in the fully adjusted models. We observed small differences between the three models where we included different sets of confounding variables.

When we investigated block apartment residents alone, the prevalence of VTE differed significantly across floor levels (*p* = 0.012), being the highest at the 6th–10th floor (9%) (Table 4). The prevalence was also the highest at either of the two highest floor levels for stroke and IC, but the tests for association did not reach statistical significance (stroke, *p* = 0.481; IC, *p* = 0.051).

The associations found between residing on higher floors and CVD among all residents (Table 3) were substantially attenuated when we investigated block apartment residents alone (Table 5). The association between residing on the 6th–10th floor and prevalence of VTE was the only crude association that remained statistically significant (OR: 1.615; 95% CI: 1.069–2.439). This association was also close to significant in the fully adjusted model (OR: 1.517; 95% CI: 0.992–2.321). When collapsing the two highest floor categories into one category (≥6th floor) (analysis not shown), residents on these floor levels had significantly increased odds of having experienced VTE (OR: 1.504; 95% CI: 1.007–2.247; *p* = 0.046). The estimates of the odds ratio of having experienced stroke and IC among residents on the 11th floor (*N* = 80) indicated positive associations, also in fully adjusted analyses, but were not statistically significant (OR: 1.215; 95% CI: 0.494–2.991 (stroke); OR: 1.418; 95% CI: 0.637–3.153 (IC)).

A protective crude association appeared when we investigated block apartment residents separately (Table 5): residing on the 4th-5th floor was associated with significantly lower odds of having experienced IC compared to those in the lowest floor category (OR: 0.633; 95% CI: 0.444–0.904), but the association did not remain significant in the adjusted analyses (OR: 0.796; 95% CI: 0.551–1.151). The prevalence of CVD among block apartment residents showed a U-shaped pattern with a minimum at the 4th-5th floor (Table 4). We did not find evidence of an overall linear trend of increasing CVD prevalence by increasing floor level among block apartment residents (*p* > 0.31 in all instances).

When we included the interaction terms between floor level and period of residence (1–10 years versus >10 years), we did not find evidence that time lived at the different floor levels modified any of the associations (*p* > 0.25).

## 4. Discussion

### 4.1. Summary of Main Findings

In this study, we found significant crude associations between floor levels and the three outcomes. The association disappeared for stroke when we included the confounding variables but remained statistically significant for participants with IC and a history of VTE living in upper floors compared to the lowest level (0-1st floor). We found statistically significant linear trends (gradients) for all outcomes in adjusted model, but no such trends were observed among individuals living in block apartments. For block apartment residents, the effect measures were attenuated and not statistically significant, except for higher odds of history of VTE in residents of the 6th floor or higher when we erased the highest category border at the 10th floor.

### 4.2. Previous Studies and Possible Explanations

Our results are in opposition to the only other previous study on the association between floor level and CVD that we have found. Panczak and coworkers (2013) reported a negative association between floor level and stroke mortality, as well as total CVD mortality, in a longitudinal study of the entire adult Swiss population of residents in buildings with four floors or more. The present study differs from the Swiss study by investigating a different population. In addition, our study is cross-sectional; we control for relevant health behaviors; we investigate additional outcomes such as VTE and IC; and we investigate the association between floor level and CVD for all types of housing and for block apartment residents only.

### 4.3. Air Pollution, Environmental Noise, and Physical Activity (Use of Stairs)

Vertical variations of air pollution [24, 25], environmental noise [26], and physical activity by use of stairs were suggested by Panczak and coworkers (2013) as possible explanations for floor level effects on CVD, as such mechanisms would explain the negative associations of their study. In the current study, no statistical test can support a negative association, despite the fact that both environmental noise and air pollution are known to be widespread hazards also in Oslo [27, 28]. We were not able to adjust our results depending on the residential area exposure of air pollution and environmental noise, or the presence and usage of lifts in the buildings. Thus, effects of air pollution, environmental noise, or use of stairs may still exist at a local level. A tendency of a decreasing CVD prevalence could be seen from the prevalence estimates among block apartment residents, which decreased from ground floor and up to the 5th floor. There was also a statistically significant lower odd of IC in residents on the 4th-5th floor of block apartments (crude association), but it disappeared when controlling for sociodemographics.

### 4.4. Socioeconomic Status and Building Height

A large proportion of individuals with low socioeconomic status resided on higher floors. SES is a particularly important aspect with regard to the development of CVD [13], and the statistically significant trends that we found are comparable to the known gradient of increasing CVD risk by decreasing SES [29]. Thus, residual confounding by SES is one possible explanation to our findings, especially since we were not able to adjust for income and wealth. However, this interpretation has an important caveat: the distribution of SES in our study, with lower SES at higher floors, is in sharp contrast to studies from other countries, which have found higher apartment costs at higher floor levels [18, 30, 31]. This discrepancy could be due to the fact that building height is a confounder for floor level in our study and that we more precisely capture effects of a declining SES as the buildings get taller. The tallest multistory buildings (high-rises), are generally viewed as less attractive [17], a view which also is applicable in Oslo [32]. With this explanation, it makes sense that the trends disappeared once we investigated block apartment residents alone, a partial control for building height. The remaining association between residing on the 6th floor or higher and VTE history in block apartment residents may be explained by further confounding by building height, since this analysis in part compares residents of high-rises to residents of low-rises. Poor cardiovascular health could reduce income opportunities and stratify individuals of low SES into more affordable high-rises. Conversely, it may also be that living in tall buildings per se has an impact on the development of CVD.

The latter view is supported by a prospective study of elder Americans finding an unexplained increased stroke risk in individuals living in multistory buildings [19]. It is possible that the psychosocial environment of such housing environments puts a toll on the cardiovascular health, as high-rises consistently have been associated with less perceived control and poorer social relations [16, 17, 33, 34]. Psychosocial factors have recently been found to relate to the development of not only arterial CVDs, but also VTE [9], which is the CVD with the most robust results in our study. The fact that we adjusted for individual CVD risk factors, in particular health behaviors such as smoking, which also are known to explain socioeconomic differences in the incidence of CVD [13], substantiates the argument of an impact of environmental factors. Psychosocial hazards may also depend on residential floor level within multistory buildings. Six of eight studies have found poorer mental health on higher floor levels [16], and one study reported that upper floor levels were associated with a poorer psychosocial climate [35].

### 4.5. Atmospheric Electricity

The higher occurrence of CVD at higher floors may also fit a recent hypothesis about the impact of atmospheric electricity on the health of residents at higher floor levels [20]. The idea is based on the fact that the electric potential of the air, in general, increases as a function of height above the ground [36]. This property of air is known as the vertical potential gradient (VPG) of the global electric circuit (GEC) [36]. Studies have found that equalizing the electric potential between the surface and Earth and the human body (earthing) improves inflammatory markers [37, 38] and blood viscosity [39, 40]. Inflammation and blood viscosity are important factors in the pathogenesis of atherosclerosis and atherothrombosis [41, 42] and hypertension [43, 44], respectively. Furthermore, a positive VPG [36] implies that the air contains increasingly higher ratios of positive air ions compared to negative air ions, as a function of distance from ground level. Animal experiments have found effects of positive air ions on increased blood coagulation and blood viscosity ([45–48] in [49, p. 124]). However, an impact of these vertically distributed constituents of atmospheric electricity on buildings seems unlikely since atmospheric physicists underline the notion that the GEC is easily blocked [50, 51] and that modern buildings specifically are believed to result in a “Faraday cage” effect [52]. In addition, numerous natural and anthropogenic factors may alter the strength and even direction (sign) of the VPG [36, 50, 51]. These theoretical shortcomings make the hypothesis debatable.

### 4.6. The Temporal Aspect (Causal Criteria)

The statistically nonsignificant interaction between period of residence (1–10 years versus more than 10 years) and floor level indicates that exposure time to floor level is not associated with more CVDs (causal criteria). However, we cannot rule out a causal link. The time measure is rough, and we cannot ascertain that the floor level of the participants' previous residence was different. In fact, settlement patterns do show consistencies; it is, for instance, common to settle close to people one considers similar in terms of SES [53].

### 4.7. Late-Look Bias

The estimated associations between floor level and stroke were consistently lower than for the other CVD outcomes. It is possible that a late-look bias [22] due to relocation may have attenuated the association between floor level and stroke. Stroke victims on higher floor levels may move to lower floors before participating in the survey. Stroke is known to be a disabling disease where half of the surviving stroke victims do not recover without residual disabilities [54], and the event of a stroke has been found to increase the odds of relocation [55], which may also imply relocation to lower floor levels to ease the daily activities.

### 4.8. Generalizability (Ecological Validity)

Several factors may affect the generalizability of the results across time and place. Selection into floor level and type of housing may depend strongly on social, economic, and cultural environment (e.g., the housing market and housing policies) which change over time and differ greatly across countries and even cities. Oslo, which is the capital of Norway, has its own distinctive characteristics with regard to distribution of type, standard, and age of buildings, which may affect the public perception of the social status of different housing types. For instance, the tallest buildings in Oslo were built in the 1960s and 1970s and have a monotonous and “brutal” design [32]. In Switzerland, on the other hand, high-rises are modern buildings associated with status and they have “prime locations” [18]. “Prime locations” possibly imply closeness to major roads and junctions and thus also more environmental noise and air pollution. This could be less likely in Oslo.

### 4.9. Strengths and Limitations

There are two major strengths in this study. The study is based on a large representative sample of an urban population, and we were able to include confounding factors known to be associated with risk of CVD. Nevertheless, the study would have improved if we had been able to include variables on income and the height of the block apartment buildings. In addition, it is still possible that an unknown variable confounds the association between residence floor level and the outcome variables.

An important limitation is the cross-sectional design, which cannot ascertain causality. The design can also be affected by a late-look bias [22], as discussed above. The outcome variables are self-reported and are not validated against other medical registries. The validity is particularly a concern regarding VTE as the disease inquiry utilized also includes the term “phlebitis.” It is also known that the majority of VTE cases are caused by major provoking factors (e.g., surgery and immobilisation) [5], which can act as confounders. We used sensitive case definitions, but this will likely attenuate our results. Self-reported measures are in general of less quality than objectively assessed information retrieved from national registries. In this study, a large proportion (about 11%) of those who reported to live on the 11th floor or higher reported their buildings to be detached houses/villas, which is not likely. The high nonresponse rate (above 50%) poses a threat to the internal validity if not responding relates to both floor level and CVD. However, Søgaard and coworkers investigated the impact of self-selection in the Oslo Health Study, which is the health study HELMILO is based on. They found that the prevalence estimates were not affected by self-selection according to sociodemographic variables [56]. A final important limitation is that we started with a relatively large sample size, but when we restricted our analysis to residents of block apartments, the sample size was substantially reduced. This could produce a type II error. The loss due to missing data was relatively small (10.4%) and we decided to report results from complete-case analyses only.

## 5. Conclusions and Implications 

In this study, we found that floor level was positively associated with CVD history in adult inhabitants of Oslo. The findings may point to residual confounding by building height and socioeconomic status and underline a need to understand possible associations between residing in tall buildings and CVD. A causal effect of residing on higher floor levels per se seems less likely but could stem from a poorer psychosocial environment on higher floor levels. The alternative explanation of an impact of atmospheric electrical parameters may also be considered. We found no statistically significant negative associations between floor level and CVD, which questions the impact of higher levels of air pollution, environmental noise, or less use of stairs at lower floor levels on CVD, but the study design limits the ability to identify such effects. As the present study contradicts a previous study from Switzerland, more studies are needed to fully understand the association between floor level and CVD and to disentangle possible causal mechanisms.

## Figures and Tables

**Table 1:** Sample characteristics dependent on floor level.

	Floor level					Total (*N* = 11.169)
	Basement and 1st floor (*N* = 3.633)	2nd-3rd floor (*N* = 5.732)	4th-5th floor (*N* = 1.281)	6th–10th floor (*N* = 424)	≥11th floor (*N* = 99)	
*Housing characteristics*						
Type of housing						
Block apartment/terraced flat	35.0	41.8	94.6	97.4	80.8	48.1
Detached house/villa	36.2	27.3	0.3	0.5	11.1	25.9
Un/semidetached house	26.0	27.8	0.5	0.9	4.0	22.8
Other residences	2.8	3.1	4.5	1.2	4.0	3.1
Period of residence						
1–10 years	37.7	39.6	49.4	48.6	31.3	40.4
>10 years	62.3	60.4	50.6	51.4	68.7	59.6
*Sociodemographics*						
Age (year of birth)						
1924-25	9.4	8.5	6.6	13.2	18.2	8.9
1940-41	28.8	23.2	28.1	32.5	31.3	26.0
1955	20.1	22.6	19.1	16.7	21.2	21.2
1960	22.0	24.2	20.1	19.8	12.1	22.7
1970	19.7	21.4	26.0	17.7	17.2	21.2
Gender (men)	46.0	46.4	48.9	47.4	54.5	46.7
Living with someone (yes)	79.3	78.5	61.5	54.2	58.6	75.7
Country of origin						
Norway	80.8	81.7	77.7	74.3	76.8	80.6
Other western countries	6.4	6.1	6.2	7.5	2.0	6.2
Other nonwestern countries	12.8	12.2	16.2	18.2	21.2	13.2
*Socioeconomics*						
Education (in years)						
≤12	32.0	28.6	32.7	40.8	52.5	30.9
13–16	36.2	33.4	35.1	27.6	31.3	34.3
>16	31.8	38.0	32.2	31.6	16.2	34.9
Occupational status						
Leader, politician	17.4	17.6	13.5	13.9	14.1	16.9
Occ. req. higher education	42.6	45.3	41.6	36.8	28.3	43.5
Office, sales, care, etc.	29.2	27.6	32.4	35.8	39.4	29.1
Blue collar and farming	10.8	9.5	12.5	13.4	18.2	10.5
*Health behaviors*						
Body mass index (BMI)						
Normal and underweight	52.5	54.5	55.0	50.7	46.5	53.7
Overweight	36.7	35.4	33.7	33.7	41.4	35.6
Obese	10.8	10.1	11.3	15.6	12.1	10.7
Physical activity (PA) level						
1 (sedentary)	9.9	9.0	11.2	14.9	15.2	9.8
2	32.0	30.4	29.7	34.2	36.4	31.0
3	33.2	35.8	38.3	37.0	26.3	35.2
4	20.5	19.7	14.7	9.9	16.2	19.0
5 (very active)	4.5	5.0	6.2	4.0	6.1	5.0
Smoking status						
Current smoker	15.5	14.5	18.8	18.2	21.2	15.5
Former smoker	38.5	37.5	36.5	41.7	28.3	37.8
Never smoked (RG)	46.0	48.0	44.7	40.1	50.5	46.7
Alcohol consumption						
Frequent	41.5	43.1	43.2	41.7	31.3	42.4
Moderate	42.1	40.2	37.5	35.8	41.4	40.4
Infrequent	16.4	16.7	19.2	22.4	27.3	17.2
Fatty fish consumption						
High	4.7	4.5	4.0	3.5	6.1	4.5
Moderate	64.2	63.7	62.8	67.7	62.6	63.9
Low	31.1	31.8	33.3	28.8	31.3	31.6
Vegetable consumption						
High	42.7	44.9	39.8	37.3	37.4	43.3
Moderate	53.8	52.5	56.8	56.1	59.6	53.6
Low	3.6	2.6	3.4	6.6	3.0	3.2
Fruit consumption						
High	44.1	46.5	44.3	39.4	32.3	45.1
Moderate	47.9	47.1	46.8	50.2	60.6	47.6
Low	8.0	6.4	8.9	10.4	7.1	7.4

All values are given in percentages.

**Table 2:** Prevalence of disease by floor level and chi-square tests of difference (all types of housing).

	Total (*N* = 11169)	Basement and 1st floor (*N* = 3633)	2nd-3rd floor (*N* = 5732)	4th-5th floor (*N* = 1281)	6th–10th floor (*N* = 424)	≥11th floor (*N* = 99)	*p* value
Stroke	4.6% (*N* = 514)	4.8% (*N* = 173)	4.0% (*N* = 230)	5.4% (*N* = 69)	7.3% (*N* = 31)	11.1% (*N* = 11)	<0.001^*∗∗∗*^
Venous thromboembolism (VTE)	4.9% (*N* = 548)	4.6% (*N* = 167)	4.8% (*N* = 275)	4.5% (*N* = 58)	9.0% (*N* = 38)	10.1% (*N* = 10)	<0.001^*∗∗∗*^
Intermittent claudication (IC)	4.8% (*N* = 540)	4.9% (*N* = 179)	4.6% (*N* = 265)	4.4% (*N* = 56)	6.4% (*N* = 27)	13.1% (*N* = 13)	0.001^*∗∗*^

^*∗∗*^ *p* < 0.01.

^*∗∗∗*^ *p* < 0.001.

**Table 3:** Results from logistic regression models including all types of housing (*N* = 11169). The figures represent odds ratios (OR) including 95% confidence intervals (CI) for the different outcomes. The category basement and 1st floor is reference category.

	Basement and 1st floor (*N* = 3633)	2nd-3rd floor (*N* = 5732)		4th-5th floor (*N* = 1281)		6th–10th floor (*N* = 424)		≥11th floor (*N* = 99)		*p*-trend
		OR	95% CI	OR	95% CI	OR	95% CI	OR	95% CI	
*Stroke*										
Model 0	1 (ref.)	0.836	(0.683–1.023)	1.139	(0.855–1.517)	1.578	(1.061–2.345)	2.500	(1.311–4.766)	<0.001^*∗∗∗*^
Model 1	1 (ref.)	0.912	(0.742–1.122)	1.206	(0.895–1.623)	1.281	(0.849–1.932)	1.936	(0.990–3.785)	0.001^*∗∗*^
Model 2	1 (ref.)	0.915	(0.744–1.126)	1.193	(0.886–1.608)	1.240	(0.821–1.873)	1.846	(0.945–3.605)	0.002^*∗∗*^
Model 3	1 (ref.)	0.907	(0.736–1.118)	1.172	(0.868–1.583)	1.183	(0.779–1.795)	1.876	(0.953–3.691)	0.002^*∗∗*^
*Venous thromboembolism (VTE)*										
Model 0	1 (ref.)	1.046	(0.859–1.274)	0.984	(0.725–1.336)	2.043	(1.415–2.951)	2.332	(1.191–4.566)	<0.001^*∗∗∗*^
Model 1	1 (ref.)	1.129	(0.924–1.379)	1.052	(0.770–1.439)	1.814	(1.242–2.647)	1.961	(0.985–3.906)	0.007^*∗∗*^
Model 2	1 (ref.)	1.138	(0.932–1.391)	1.046	(0.765–1.431)	1.768	(1.210–2.585)	1.873	(0.940–3.733)	0.012^*∗*^
Model 3	1 (ref.)	1.143	(0.935–1.398)	1.043	(0.761–1.429)	1.720	(1.174–2.518)	1.873	(0.939–3.738)	0.015^*∗*^
*Intermittent claudication (IC)*										
Model 0	1 (ref.)	0.935	(0.770–1.136)	0.882	(0.649–1.199)	1.312	(0.864–1.993)	2.917	(1.597–5.327)	<0.001^*∗∗∗*^
Model 1	1 (ref.)	1.007	(0.825–1.228)	0.950	(0.639–1.303)	1.111	(0.722–1.708)	2.410	(1.288–4.509)	0.003^*∗∗*^
Model 2	1 (ref.)	1.015	(0.832–1.238)	0.940	(0.685–1.290)	1.083	(0.703–1.668)	2.292	(1.226–4.283)	0.005^*∗∗*^
Model 3	1 (ref.)	1.020	(0.835–1.246)	0.951	(0.692–1.308)	1.067	(0.691–1.647)	2.318	(1.237–4.345)	0.005^*∗∗*^

Model 0: crude associations.

Model 1: age, gender, living with someone, and country of birth (sociodemographics).

Model 2: Model 1 + education and occupational status (SES).

Model 3: Model 2 + BMI, PA, smoking status, alcohol consumption, and diet (health behaviors).

^*∗*^ *p* < 0.05.

^*∗∗*^ *p* < 0.01.

^*∗∗∗*^ *p* < 0.001.

**Table 4:** Prevalence of disease by floor level and chi-square tests of difference (block apartment residents only).

	Total (*N* = 5374)	Basement and 1st floor (*N* = 1271)	2nd-3rd floor (*N* = 2398)	4th-5th floor (*N* = 1212)	6th–10th floor (*N* = 413)	≥11th floor (*N* = 80)	*p* value
Stroke	5.6% (*N* = 303)	5.9% (*N* = 75)	5.4% (*N* = 129)	5.2% (*N* = 63)	7.3% (*N* = 30)	7.5% (*N* = 6)	0.481
Venous thromboembolism (VTE)	5.9% (*N* = 316)	5.7% (*N* = 73)	6.0% (*N* = 145)	4.5% (*N* = 54)	9.0% (*N* = 37)	8.8% (*N* = 7)	0.012^*∗*^
Intermittent claudication (IC)	5.8% (*N* = 314)	6.6% (*N* = 84)	6.0% (*N* = 144)	4.3% (*N* = 52)	6.3% (*N* = 26)	10.0% (*N* = 8)	0.051

^*∗*^ *p* < 0.05.

**Table 5:** Results from logistic regression models (block apartment residents only (*N* = 5374)). The figures represent odds ratios (OR) including 95% confidence intervals (CI) for the different outcomes. The category basement and 1st floor is reference category.

	Basement and 1st floor (*N* = 1271)	2nd-3rd floor (*N* = 2398)		4th-5th floor (*N* = 1212)		6th–10th floor (*N* = 413)		≥11th floor (*N* = 80)		*p*-trend
		OR	95% CI	OR	95% CI	OR	95% CI	OR	95% CI	
*Stroke*										
Model 0	1 (ref.)	0.907	(0.676–1.215)	0.874	(0.619–1.234)	1.249	(0.805–1.937)	1.293	(0.545–3.068)	0.363
Model 1	1 (ref.)	0.921	(0.682–1.243)	1.030	(0.723–1.467)	1.151	(0.734–1.807)	1.170	(0.482–2.842)	0.377
Model 2	1 (ref.)	0.930	(0.689–1.257)	1.054	(0.739–1.503)	1.166	(0.741–1.833)	1.138	(0.469–2.764)	0.354
Model 3	1 (ref.)	0.935	(0.689–1.269)	1.076	(0.750–1.544)	1.134	(0.715–1.798)	1.215	(0.494–2.991)	0.316
*Venous thromboembolism (VTE)*										
Model 0	1 (ref.)	1.056	(0.790–1.411)	0.765	(0.533–1.098)	1.615	(1.069–2.439)	1.574	(0.700–3.540)	0.715
Model 1	1 (ref.)	1.082	(0.806–1.452)	0.876	(0.607–1.263)	1.568	(1.030–2.386)	1.475	(0.645–3.371)	0.516
Model 2	1 (ref.)	1.086	(0.809–1.459)	0.895	(0.620–1.293)	1.563	(1.025–2.383)	1.446	(0.632–3.311)	0.484
Model 3	1 (ref.)	1.091	(0.810–1.467)	0.900	(0.621–1.303)	1.517	(0.992–2.321)	1.440	(0.626–3.310)	0.491
*Intermittent claudication (IC)*										
Model 0	1 (ref.)	0.903	(0.684–1.192)	0.633	(0.444–0.904)	0.949	(0.603–1.496)	1.570	(0.732–3.368)	0.573
Model 1	1 (ref.)	0.905	(0.680–1.204)	0.741	(0.515–1.066)	0.881	(0.553–1.405)	1.424	(0.646–3.139)	0.551
Model 2	1 (ref.)	0.918	(0.690–1.222)	0.761	(0.529–1.096)	0.894	(0.559–1.428)	1.380	(0.626–3.039)	0.519
Model 3	1 (ref.)	0.945	(0.708–1.262)	0.796	(0.551–1.151)	0.886	(0.552–1.423)	1.418	(0.637–3.153)	0.549

Model 0: crude associations.

Model 1: age, gender, living with someone, and country of birth (sociodemographics).

Model 2: Model 1 + education and occupational status (SES).

Model 3: Model 2 + BMI, PA, smoking status, alcohol consumption, and diet (health behaviors).
